# Temporal trends in abusive alcohol consumption and their projections for 2030 in Brazilian capitals

**DOI:** 10.1590/1980-549720250057

**Published:** 2026-01-12

**Authors:** Deborah Carvalho Malta, Paula Carvalho de Freitas, Crizian Saar Gomes, Guilherme Augusto Veloso, Regina Tomie Ivata Bernal, Alanna Gomes da Silva, Filipe Malta Santos, Sther Luna Abras dos Santos, Paulo Ferrinho

**Affiliations:** I Universidade Federal de Minas Gerais, School of Nursing - Belo Horizonte (MG), Brazil.; II Ministério da Saúde, Secretaria de Vigilância em Saúde e Ambiente - Brasília (DF), Brazil.; III Universidade Federal de Minas Gerais, School of Medicine - Belo Horizonte (MG), Brazil.; IV Universidade Federal Fluminense - Niterói (RJ), Brazil.; V Faculdade de Ciências Médicas de Minas Gerais - Belo Horizonte (MG), Brazil.; VI Universidade Nova de Lisboa, Institute of Hygiene and Tropical Medicine - Lisboa, Portugal.

**Keywords:** Binge drinking, Sustainable development, Health surveys, Time factors, Chronic disease

## Abstract

**Objective:**

To analyze the temporal trend of the prevalence of abusive alcohol consumption in the Brazilian adult population residing in the country’s capitals between 2006 and 2023, and to estimate projections for 2030.

**Methods:**

Time-series study using data from the Surveillance System for Risk and Protective Factors for Chronic Diseases by Telephone Survey. Abusive alcohol consumption was defined as the intake of five or more drinks for men and four or more for women in a short period of time. The joinpoint regression model was used to identify trends, and exponential smoothing was applied to generate projections up to 2030.

**Results:**

We observed an increasing trend in abusive alcohol consumption in the total population (from 15.6% in 2006 to 20.8% in 2023) and among women, across most age groups - except those aged 18-24 and 45-54 years -, among individuals with 12 or more years of education, and among racial groups identified as white, Black, and mixed-race. Among women, we observed an increase in 23 capitals. Considering the trends between 2015 and 2023, the 10% reduction target by 2030 is unlikely to be achieved.

**Conclusion:**

The increase in alcohol consumption among women and the failure to meet the reduction target highlight the need to advance policies and programs to curb alcohol consumption.

## INTRODUCTION

Alcohol is a toxic and psychoactive substance that can cause dependence among its consumers. In many societies, alcoholic beverages are a frequent part of social interactions, and it is easy to ignore or disregard the social and health damage caused by their consumption^1^. According to the World Health Organization (WHO), alcohol consumption contributes to 2.6 million deaths every year worldwide, of which 1.6 million are caused by chronic noncommunicable diseases (NCDs), 700 thousand deaths from injuries, and 300 thousand deaths from communicable diseases^1^.

The harmful use of alcoholic beverages accounts for 4.7% of the global burden of diseases, being 6.9 and 2% among men and women, respectively. Alcohol is the main risk factor for premature mortality and disability among people aged 20 to 39 years, accounting for 13% of all deaths in this age group. Disadvantaged and especially vulnerable populations have higher rates of death and hospitalization from alcohol consumption^1^.

In Brazil, alcohol consumption was the seventh risk factor in 2021 for loss of disability-adjusted life years (DALYs), leading to 2,710,367 million (3.45%) DALYs^2^.

Among the individuals with abusive consumption of alcoholic beverages in 2020, 59.1% aged between 15 and 39 years and 76.9% were men^3^.Although the prevalence of consumption is higher among men, there have been changes in this pattern, and women have increased their consumption, tending to the convergence among prevalence rates by sex^4^. This change may be related to the increase in marketing aimed at women by the alcohol industry, aiming to expand its consumption base, a phenomenon that has been described as a commercial determinant of health, or the industry’s pressure to change local consumption patterns and increase its profitability^4^ ^,^ ^5^ ^,^ ^6^ ^,^ ^7^.

The industry acts on several fronts and strategies, among them in the cultural environment, through advertising and action on social media, seeking engagement, disseminating fake news, aiming to establish new behavior parameters. Certain countries and regions, such as low- and middle-income countries, face greater pressure from transnational actors^8^.

In order to reduce abusive alcohol consumption, several global and national initiatives have been adopted. In May 2010, the World Health Assembly approved the Global Strategy to Reduce the Harmful Use of Alcohol^1^; in 2018, the SAFER initiative was launched by the WHO, which launched in Brazil in 2019^1^ ^,^ ^2^. These initiatives aim to support countries in the implementation of high-impact, cost-effective interventions that are proven to reduce the damage caused by alcohol consumption^1^.

The monitoring of alcohol consumption is part of the targets of the Agenda for Sustainable Development^9^, which aims at a 10% reduction in alcohol consumption by 2030. In Brazil, the Strategic Action Plan to Tackle Noncommunicable Diseases also proposes similar goals; however, no reduction has been observed in the country^10^. Therefore, monitoring alcohol consumption in order to identify the groups with greater exposure can contribute to the development of health policies and programs to tackle the issue.

In this sense, in this study, we aimed to analyze the temporal trend of the prevalence of abusive alcohol consumption in the Brazilian adult population living in Brazilian capitals between 2006 and 2023 and to estimate the projections for 2030.

## METHODS

### Study design

Time-series study using data from the Surveillance System for Risk and Protective Factors for Chronic Diseases by Telephone Survey (*Vigilância de Fatores de Risco e Proteção para Doenças Crônicas por Inquérito Telefônico* - Vigitel), between 2006 and 2023.

### Context

Vigitel is a population-based telephone survey conducted annually by the Brazilian Ministry of Health. It collects information on NCDs as well as on the main risk and protective factors related to these diseases in the Brazilian adult population (≥18 years of age). Until 2021, sampling procedures aimed to obtain, in each capital of the 26 Brazilian states and in the Federal District, probability samples of the population of adults living in households that had at least one landline. In 2023, interview through mobile phones was incorporated. In the editions between 2006 and 2019, a sample size of at least two thousand adults per city was established. However, in the years 2020 and 2021, due to the difficulties imposed by the COVID-19 pandemic on data collection, the sample size was reduced to about one thousand individuals per city. For the year 2023, a further reduction was necessary, setting a minimum of 800 interviews in each of the localities. Moreover, the rapid deterioration of landline coverage in the country motivated half of the interviews to be conducted by mobile phone in order to allow the estimation of good quality data (with a final sample of 400 interviews by landline and 400 by mobile phone in each locality)^11^ ^,^ ^12^.

The Vigitel sampling process takes place in two stages. The first stage is the draw of telephone lines per city. Next, the lines drawn in each city are drawn once again and divided into replicas (200 lines for landlines and 500 for mobile phones). The second stage (for landline cases only) consists of the draw of one of the adults (≥18 years of age) for the interview^13^.

As the sample of adults interviewed by Vigitel was extracted from the telephone line register (residential landlines and mobile phones), allowing population inferences only for the adult population that had a telephone in each of the localities, weights (called post-stratification weights) are attributed to each individual to match the estimated sociodemographic composition for the Vigitel sample in each city to the sociodemographic composition estimated for the total adult population of the same city^12^. The post-stratification weight of each individual in the Vigitel sample was calculated by the raking method^12^ ^,^ ^13^.

More information on the sampling and selection process can be found in the annual reports for results dissemination^13^.

### Variables

In the present study, abusive alcohol consumption was analyzed according to the following questions:

• For men: In the last 30 days, did you have five or more drinks on a single occasion?;
• For women: In the last 30 days, did you have four or more drinks on a single occasion?

The analyzed indicator, “abusive alcohol consumption,” constitutes the consumption of an excessive volume of alcoholic beverages in a short period of time, namely five or more drinks for men and four or more drinks for women^2^ ^,^ ^13^.

### Data analysis

The prevalence of alcohol abuse was calculated for the total population and it was stratified according to sex (women and men); age group (18 to 24; 25 to 34; 35 to 44; 45 to 54; 55 to 64; and 65 years or over); level of education (0 to 8; 9 to 11; and 12 years or over of formal education); race/skin color (white, Black, Asian, mixed-race, and Indigenous); regions (North, Northeast, Midwest, Southeast, and South); and Brazilian capitals.

The analysis of the temporal trends of alcohol abuse was performed using the joinpoint regression model, seeking to identify changes in data behavior throughout the 2006-2023 period. For evaluating the trend during the period, the average annual percent change (AAPC) was used. The existence of a significant trend was considered when AAPC was statistically different from 0, that is, when the Student’s t-test p-value was equal to or less than 5%, rejecting the null hypothesis of trend absence. The direction of the trend was interpreted based on the AAPC signs: positive values indicate increase, while negative values suggest decrease.

According to the targets set by the Sustainable Development Goals to reduce alcohol consumption by 10% between 2015 and 2030, a projection for 2030 for the total population and according to sex was performed using the exponential smoothing model applied to all observed data.

All analyses considered the weighting factors, taking into account the unequal probability that individuals living in households with a higher number of landlines or fewer residents had to participate in the sample.

For data analysis, RStudio and Joinpoint software were used.

### Ethical aspects

The informed consent form was verbally obtained via telephone contact with the interviewees. Vigitel was approved by the National Commission of Ethics in Research with Human Beings of the Ministry of Health (CAAE: 65610017.1.0000.0008), opinion No. 4.324.071.

## Data Availability Statement:

The data used in this study are public and can be accessed at: https://svs.aids.gov.br/download/Vigitel/.

## RESULTS

Between 2006 and 2023, a total of 806,169 individuals were interviewed, distributed per year as follows: 2006 (54,369); 2007 (54,251); 2008 (54,353); 2009 (54,367); 2010 (54,339); 2011 (54,144); 2012 (45,448); 2013 (52,929); 2014 (40,853); 2015 (54,174); 2016 (53,210); 2017 (53,034); 2018 (52,395); 2019 (52,443); 2020 (27,077); 2021 (27,093); and 2023 (21,690).

There was an increase in the prevalence of abusive alcohol consumption among the population living in Brazilian capitals, from 15.7% in 2006 to 20.8% in 2023. When analyzing trends by sex, we verified a significant increase among women - from 7.8% in 2006 to 15.2% in 2023 - and stability among men, with a prevalence of 25% in 2006 and 27.3% in 2023. Abusive alcohol consumption also increased in all age groups, except among young people aged 18 to 24 years and adults aged 45 to 54 years. Among people with 12 years or over of formal education, the prevalence rose from 18.1 to 24%. We also observed an increase in the following races/skin colors: white (14.7 to 21%), Black (18.8 to 23.2%), and mixed-race (16.1 to 20.3%), and a decrease among Asian (16.1 to 14.3%). There was an increase in prevalence in the Midwest (15.5 to 23.6%), Northeast (18.4 to 21.4%), Southeast (14.3 to 20.8%), and South (13.2 to 20.7%) regions (Table 1).

**Table 1.** Temporal trend of prevalence of abusive alcohol consumption according to sex, age group, level of education, and race. Surveillance System for Risk and Protective Factors for Chronic Diseases by Telephone Survey (Vigitel), 2006 to 2023.

	2006	2007	2008	2009	2010	2011	2012	2013	2014	2015	2016	2017	2018	2019	2020	2021	2023	AAPC
Total	15.7	16.5	17.2	18.4	18.1	16.5	18.4	16.4	16.5	17.2	19.1	19.1	17.9	18.8	20.9	18.3	20.8	1.1*
Sex																		
Men	25.0	25.5	26.1	28.3	27.0	25.3	27.9	24.2	24.8	25.3	27.3	27.1	26.0	25.3	26.6	25.0	27.3	0.0
Women	7.8	8.7	9.6	10.0	10.5	9.0	10.3	9.7	9.4	10.2	12.1	12.2	11.0	13.3	16.0	12.7	15.2	3.3*
Level of education (years)																		
0 to 8	13.6	13.9	14.5	14.5	14.0	13.4	15.0	12.8	12.3	13.2	14.2	13.8	13.0	12.4	15.0	11.7	14.4	-0.5
9 to 11	17.0	18.5	19.2	19.8	19.6	17.5	19.4	17.5	18.4	18.1	19.2	20.2	19.1	20.0	22.5	19.4	22.1	0.7
≥12	18.1	18.7	19.5	23.7	22.9	20.0	22.0	19.7	19.5	20.9	24.0	22.8	21.2	23.1	23.8	22.5	24.0	1.1*
Age group (years)																		
18 to 24	19.0	22.3	21.3	23.3	22.0	20.2	21.8	19.0	18.2	20.2	22.1	23.8	23.0	25.8	25.0	19.3	21.4	0.7
25 to 34	21.7	21.6	22.1	23.9	24.1	21.3	24.7	22.7	23.2	23.5	25.8	27.7	24.2	26.3	30.9	25.5	29.8	1.6*
35 to 44	17.6	16.5	19.3	20.0	19.8	18.2	20.0	17.5	18.0	19.4	21.2	22.2	21.7	20.9	21.5	20.0	24.7	1.5*
45 to 54	13.2	14.3	15.2	16.8	15.9	14.8	16.6	15.0	15.1	15.5	18.2	15.8	14.7	15.8	18.2	17.9	21.1	1.3
55 to 64	6.6	9.6	10.3	10.4	10.7	10.6	11.9	10.5	11.0	11.0	12.6	10.3	11.0	11.2	13.8	13.2	11.4	3.3*
≥65	2.5	2.7	3.3	4.1	4.4	4.5	5.0	4.0	3.8	3.7	4.6	3.0	4.1	4.1	5.7	5.8	5.4	5.8*
Race/skin color																		
White	14.7	15.2	15.8	18.1	17.4	15.4	17.8	15.8	15.7	16.6	18.1	18.9	16.5	18.0	20.7	16.9	21.0	1.39*
Black	18.8	21.5	21.7	23	21.1	20.6	21.7	19.5	20.6	20.3	25	22	21.7	24	25.5	24	23.2	1*
Asian	16.1	17.0	17.8	18.2	18.3	14.2	16.4	17.0	17.3	16.6	12.7	10.6	14.0	14.2	11.4	10.0	14.3	-2.6*
Mixed-race	16.1	15.1	8.8	12.3	5.3	16.8	18.5	17.2	17.0	17.6	20.0	19.6	19.1	19.0	21.0	18.5	20.3	4*
Indigenous	8.7	44.1	13.2	17.8	21.9	20.8	26.1	16.3	12.5	23.9	20.0	20.7	15.0	18.1	20.0	14.4	23.8	0.3
Region																		
Midwest	15.5	16.7	17.3	18.6	18.7	15.0	19.3	17.1	18.5	20.8	22.0	23.7	19.7	21.8	24.3	21.1	23.6	2.4*
Northeast	18.4	19.2	19.9	20.8	20.8	19.2	20.5	18.0	16.7	17.2	20.1	19.4	18.7	19.0	20.4	18.8	21.4	0.8*
North	17.2	17.3	19.7	18.4	18.3	16.0	16.6	14.5	14.8	15.1	17.0	16.5	16.7	16.7	15.7	16.5	16.1	0.1
Southeast	14.3	15.2	16.0	17.6	16.9	15.9	18.1	16.2	16.5	17.2	18.7	18.5	17.7	18.8	21.8	18.3	20.8	1.7*
South	13.2	14.5	12.8	15.4	15.3	14.1	15.2	13.4	15.4	14.1	16.3	17.7	15.7	16.6	19.5	15.1	20.7	1.8*

AAPC: Average annual percent change; *significant p-value (<0.05).

In Tables 2 and 3, we show the trend of alcohol consumption stratified by sex in the capitals. Among women, in almost all capitals there was an increasing trend, except Belém (state of Pará), Macapá (state of Amapá), Manaus (state of Amazonas), and Maceió (state of Alagoas), which remained stable (Table 2). Conversely, among men, there was a decrease in consumption in five capitals - Belém, Manaus, Natal (state of Rio Grande do Norte), Recife (state or Pernambuco), São Luís (state of Maranhão) - and an increase in only two capitals - Federal District and São Paulo (state of São Paulo) (Table 3).

**Table 2.** Temporal trend of the prevalence of abusive alcohol consumption among women, per Brazilian regions and capitals. Surveillance System for Risk and Protective Factors for Chronic Diseases by Telephone Survey (Vigitel), 2006 to 2023.

	Year																	AAPC
	2006	2007	2008	2009	2010	2011	2012	2013	2014	2015	2016	2017	2018	2019	2020	2021	2023	
Location																		
North	7.7	7.4	9.2	8.6	8.1	7.9	8.2	7.0	6.6	6.5	8.5	9.7	9.2	9.4	9.6	10.9	10.3	1.8*
Belém	8.6	7.6	9.8	10.6	9.0	10.3	11.4	8.0	8.5	5.9	8.5	11.4	9.0	9.9	9.1	9.8	10.1	0.4
Boa Vista	6.4	8.7	8.5	9.6	8.8	9.3	7.8	7.2	6.1	8.3	11.9	11.1	10.0	9.5	12.8	12.8	13.5	3.2*
Macapá	8.4	9.0	5.3	9.4	9.5	8.6	7.7	8.7	7.1	8.1	9.2	8.6	10.5	6.9	9.5	13.7	9.9	1.7
Manaus	6.8	6.5	9.5	6.0	5.9	5.1	5.1	4.6	4.5	5.2	7.7	8.0	8.1	8.7	8.2	9.8	8.4	0.8
Palmas	9.9	8.9	9.6	10.1	12.1	9.7	11.0	10.7	8.9	11.1	13.1	11.8	12.9	17.4	12.4	13.0	13.6	2.6*
Porto Velho	7.3	7.3	10.0	10.9	10.1	9.0	10.7	10.6	9.0	8.0	7.5	10.6	8.9	9.3	12.4	14.4	13.0	4.4*
Rio Branco	7.3	7.3	8.0	7.7	8.5	7.0	6.3	6.3	5.5	8.0	7.2	8.6	11.2	7.7	10.9	10.2	12.1	2.7*
Northeast	9.0	10.2	10.5	10.7	11.9	10.9	11.0	10.1	8.7	9.7	12.4	11.6	11.7	12.9	14.5	13.2	14.6	3.1*
Aracaju	8.2	7.6	9.3	8.3	11.9	9.2	9.3	11.5	11.0	9.1	13.6	13.2	11.4	12.7	15.4	11.2	16.8	3.7*
Fortaleza	7.5	9.2	6.8	8.2	8.0	7.5	6.5	7.3	6.6	7.3	8.0	9.9	9.0	12.2	9.9	13.7	9.6	2.5*
João Pessoa	5.8	9.0	8.2	8.7	10.6	8.4	7.4	6.4	5.1	5.2	7.3	9.7	7.4	9.3	10.1	13.0	11.1	4.3*
Maceió	8.0	9.0	8.3	11.6	9.9	9.8	9.8	7.1	6.8	7.4	14.4	9.2	8.8	8.3	12.5	8.4	11.9	0.9
Natal	5.6	7.7	9.0	7.8	8.0	7.3	9.3	6.9	8.4	6.7	10.4	9.1	8.0	8.6	14.4	10.3	10.9	2.8*
Recife	11.9	10.3	12.0	9.3	13.5	11.9	13.2	10.3	10.8	10.3	12.9	12.1	14.0	13.5	16.9	15.2	16.6	2.6*
Salvador	11.5	13.4	15.2	15.3	16.9	16.0	16.7	13.9	11.5	15.4	18.3	15.5	16.7	18.1	21.6	15.2	21.9	2.2*
São Luís	6.7	9.4	9.4	10.1	10.6	10.6	9.4	11.9	6.4	6.9	9.9	10.8	10.5	11.3	10.6	13.7	13.0	2.2*
Teresina	9.0	9.9	9.5	10.0	11.9	9.9	10.6	12.0	8.9	10.5	12.3	9.8	11.0	11.5	11.3	10.3	12.8	1.1*
Midwest	8.3	8.7	9.1	12.8	11.2	8.6	11.5	10.2	10.4	13.6	14.4	14.3	11.2	15.2	18.8	14.8	17.0	4.1*
Federal District	8.7	8.5	10.1	14.9	12.6	8.7	13.1	9.3	11.5	15.4	15.1	16.3	11.7	17.1	18.6	16.3	20.5	4.5*
Campo Grande	8.6	8.4	8.0	10.7	10.6	7.0	9.4	12.6	7.0	7.7	11.7	10.0	9.4	13.3	19.7	15.4	14.8	3.8*
Cuiabá	7.7	9.1	9.7	9.8	10.8	10.4	11.9	11.9	11.2	14.1	14.5	14.3	12.6	10.9	16.4	12.3	16.6	3.5*
Goiânia	7.4	9.3	7.4	11.3	9.2	8.8	9.5	9.9	9.8	13.2	14.5	12.9	10.8	14.4	19.7	12.5	11.7	3.8*
Southeast	7.2	8.5	9.7	9.6	10.4	8.5	10.5	10.4	10.4	10.7	12.3	13.0	11.1	14.2	17.6	12.7	15.7	4.0*
Belo Horizonte	12.1	10.3	13.1	14.1	12.8	12.1	13.0	14.5	16.0	11.9	15.3	16.5	16.4	15.2	15.3	16.0	18.0	2.3*
Rio de Janeiro	9.6	11.8	12.1	13.3	12.6	12.5	13.3	11.0	12.2	14.4	13.7	13.2	12.7	17.6	20.4	16.6	17.7	3.0*
São Paulo	4.7	6.1	7.5	6.3	8.4	5.3	8.3	9.2	8.1	8.2	10.7	12.1	9.0	12.1	16.7	9.7	14.0	5.7*
Vitória	10.8	10.0	10.8	13.9	12.9	12.4	12.3	11.3	10.6	14.2	14.9	13.4	15.5	12.3	14.0	15.3	18.0	2.4*
South	5.8	7.5	7.1	8.0	8.3	7.7	8.2	6.8	7.8	8.6	10.8	9.8	10.5	11.1	15.4	10.5	17.4	4.9*
Curitiba	7.7	9.1	9.7	9.8	10.8	10.4	11.9	11.9	11.2	14.1	14.5	14.3	12.6	10.9	16.4	12.3	16.6	3.5*
Florianópolis	7.1	9.3	9.9	10.0	12.6	8.6	9.9	10.9	11.4	12.1	10.6	12.5	14.2	13.5	20.5	17.6	18.8	4.9*
Porto Alegre	6.7	9.4	8.8	9.2	10.2	8.6	10.5	8.7	10.2	10.0	12.5	7.3	12.6	13.0	18.0	10.5	20.7	4.2*

AAPC: Average annual percent change; *significant p-value (<0.05).

**Table 3.** Temporal trend of the prevalence of abusive alcohol consumption among men, per Brazilian regions and capitals. Surveillance System for Risk and Protective Factors for Chronic Diseases by Telephone Survey (Vigitel), 2006 to 2023.

Location	Year																	AAPC
	2006	2007	2008	2009	2010	2011	2012	2013	2014	2015	2016	2017	2018	2019	2020	2021	2023	
North	27.6	28.1	31.1	29.1	29.5	24.7	25.8	22.8	23.6	24.5	26.4	23.9	24.9	24.6	22.4	22.7	22.4	-2.6*
Belém	26.9	32.5	37.0	32.8	32	24.4	27.2	27.8	25.9	24.2	29.9	26.8	27.5	25.4	25.1	21.9	26.5	-1.6*
Boa Vista	23.9	29.4	25.4	31.1	25.5	21.8	26.9	27.7	21.1	21.6	24.5	24	30.2	28.4	25.7	23.4	28.1	0.0
Macapá	29.3	31	34.2	37.4	29.2	25.9	30.7	27.8	27.6	25.1	30.2	23.7	30.2	26	25.1	27.6	26.8	-1.3
Manaus	28.6	24.6	28.6	25.9	29.7	25	22.8	17.2	20.9	24.7	23.2	19.9	19.9	21.9	17.6	20.2	17.1	-2.6*
Palmas	28.9	30.1	31.3	27.1	31.9	29.3	32.1	29.1	25.7	27.6	34.9	33.2	32.5	28.5	34.7	32.5	26.6	0.3
Porto Velho	28	26	28.2	27.5	25.7	26.4	29.1	21.2	23.3	28.8	24.6	27.2	28.2	24.5	21.3	28.4	24.7	-0.5
Rio Branco	25.1	23.3	22.1	21.3	23.9	20.2	20.2	18.9	25.4	18.4	22.4	21.8	20.2	28.6	23.5	17.4	18.3	-0.7
Northeast	29.8	30.1	31.3	33	31.7	29.3	31.9	27.5	26.3	26.2	29.4	28.9	27.2	26.4	27.4	25.7	29.4	-0.9*
Aracaju	29.3	23.7	32	34.1	34.8	29.3	31.3	34.7	23.5	26.4	31.8	31.2	26.5	28.4	33.5	24.7	25.7	-0.6
Fortaleza	25.9	27.6	29.9	32.6	26.7	26.8	27.9	21.3	22.4	22.4	28	25.7	21.9	24.1	22.2	23.8	25.1	-1.3
João Pessoa	28.6	31.1	29.6	32.3	28	27.8	29.3	26	22.3	24.7	23.8	29.3	26.4	26.2	25.1	27.9	29.1	-0.6
Maceió	27.2	29.2	28.7	32.1	31.9	28.7	28.4	27.3	27.5	19	28.4	29.2	28.8	23.8	20.6	19	34.2	-1.2
Natal	27.5	31.3	28.9	31	34.3	28.6	29	23.9	24.9	24.3	29.7	28.5	26.1	20.8	25.8	21.4	20.8	-2.1*
Recife	32.5	30.7	28.5	29.1	35.1	28.5	30.7	28.2	22.8	27	28	29.1	28.2	25.6	26.4	24.6	27.1	-1.2*
Salvador	32.9	29.3	34	34.7	32.8	30.2	38.5	30.4	32	31	32.9	30.6	31.6	31.7	34	31.5	37.5	0.2
São Luís	30	36.5	30.7	34.8	34.4	30.7	30.7	32.6	29.4	27.6	27	31.2	25.6	24.5	29.3	23	24.7	-2.0*
Teresina	31.1	34.4	38.1	36.4	31.2	36.3	34.8	29.7	28	30.2	31.9	27.9	29.1	26.6	28.1	28.5	33	0.2
Midwest	23.3	26.3	26.6	25.2	27.2	22.3	28	24.9	27.8	29	30.6	34.3	29.3	29.4	30.4	28.3	31.1	1.5*
Federal District	22.1	26.4	25.8	23.3	28.1	21	27.5	24.5	31.9	33.7	31.1	36.4	30.7	30.9	30.8	29.7	31.9	2.1*
Campo Grande	20.6	28.1	25.5	27.4	23.9	19	28.5	23	24.9	21.5	28.2	29.9	30.8	27.4	28.9	24.7	30.2	1.3
Cuiabá	28.9	31.2	29.5	28	31.1	30.4	28.3	31.1	26.5	28.1	34.5	36.2	27.6	33.1	30.9	35	33	0.8
Goiânia	25	22.8	27.3	26.2	25.6	23.2	28.7	24.2	22.1	24.8	29.3	32	26.5	26	30.2	25.2	29.2	0.9
Southeast	22.5	23.2	23.4	27.1	24.5	24.7	27	23.1	23.6	25	26.3	24.9	25.5	24.3	26.6	25	26.8	0.6
Belo Horizonte	27.1	29.3	30	34.1	30.6	28.1	30.6	25.5	29.5	27.5	29.2	26	29.5	27.3	30.2	36.2	27.7	0.0
Rio de Janeiro	25.5	27.4	27.8	29.6	26	24.6	25.6	26	25.6	28.2	31.5	26.6	26.7	28.4	23.5	23.8	25.8	-0.3
São Paulo	19.6	19.3	19.2	24	22.2	23.9	26.9	20.8	21.2	22.5	22.7	23.6	23.8	21.2	27.6	23	27.1	1.3*
Vitória	25.4	31	28.6	29	28.9	27.5	29.4	26.6	24.6	26	30.3	27.1	30.4	26.4	29.5	32.6	29.2	0.3
South	22.1	22.6	19.4	24.1	23.4	21.6	23.4	21.2	24.3	20.5	22.7	27	21.8	23	24.2	20.5	24.5	0.4
Curitiba	20.3	21.6	18.2	22.3	21.2	22.4	21.8	20.5	20.8	19.1	23.3	28.4	22	22.4	21.6	22.7	24.6	0.9
Florianópolis	29.3	27.1	25.5	29.9	29.1	25.6	34	26.4	34.4	27.8	31.1	32.9	31	29.3	33.5	25.8	28.5	0.4
Porto Alegre	22.2	22.4	19.1	24.9	24.4	19.4	22	20.4	25.3	19.9	19.2	23.2	18.3	21.6	24.4	15.6	22.9	-0.6

AAPC: Average annual percent change; *significant p-value (<0.05).

**Figure 1.**
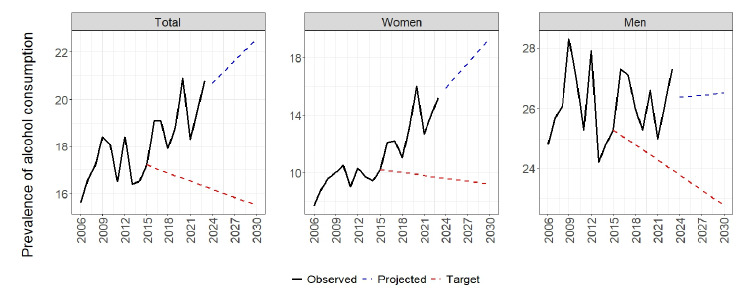
Trends and projection of the prevalence of abusive alcohol consumption in (a) adult population, (b) women and (c) men. Surveillance of Risk and Protection Factors for Chronic Diseases by Telephone Survey (Vigitel), Brazil, 2006-2030.

In Figure 1, we present the projections of the prevalence of alcohol abuse up to 2030. The target of reduction by 10%, based on the prevalence of 2015, is to reach 15.5% in 2030 for the total population, 22.8% for men, and 9.2% for women. Nonetheless, considering the trends from 2015 to 2023, the projection for 2030 is 22.2 (total population), 26.5 (men), and 19.3% (women), that is, the targets are unlikely to be achieved.

## DISCUSSION

According to our results, there is an increasing trend of abusive alcohol consumption in the total population and among women, while it remains stable among men. There was an increase in abusive consumption in the age groups of 25 to 34 years, 35 to 44 years, 55 to 64 years, and 65 years or over, as well as among the population with 12 years or over of formal education and in the races/skin colors white, mixed-race, and Black. There were variations in trends according to the analyzed regions and capitals. Among men, we identified an increase in two capitals and a decrease in five. Among women, we observed an increase in 23 capitals. Considering the trends between 2015 and 2023, the targets for reducing alcohol abuse by 10% by 2030 are unlikely to be achieved.

The increase in alcohol consumption among women and stability among men, evidenced in this study, have been reported in several studies^14^ ^,^ ^15^ ^,^ ^16^ ^,^ ^17^ ^,^ ^18^, whose authors also point to the narrowing of differences between sexes over time^4^ ^,^ ^11^ ^,^ ^18^ ^,^ ^19^. In addition, there was an increase in abusive consumption among women in most of the capitals, indicating a trend of approximation of the prevalence between men and women in the analyzed period. It is known that alcohol consumption patterns are influenced by local, regional, and cultural factors^20^. According to data from the 2019 National Survey of Health, the highest prevalence among women was recorded in the states of Bahia, Sergipe, and Mato Grosso do Sul^20^.

The increase in consumption among women has been party attributed to marketing strategies of the alcohol industry and to the role of social media, which began to direct their campaigns to the female audience^6^ ^,^ ^21^. The creation of products aimed specifically at women - such as drinks with sweet flavors, colorful packaging, and tailored drinks - has reinforced this movement^22^. The literature has widely discussed the role of commercial determinants of health in promoting consumption among women^8^, highlighting the strategic role of the industry in creating narratives that associate consumption with empowerment, freedom, and social inclusion. Moreover, sociocultural changes, such as higher level of education, greater female participation in the labor market, the adoption of new lifestyles^4^, the search for greater equality between sexes, and the incorporation of behaviors traditionally associated with the male universe^5^, can also contribute to this scenario.

Researchers show that increased alcohol consumption among women has implied several adverse effects, such as risks during pregnancy and for the baby^23^, higher incidence of breast cancer^24^, cardiovascular diseases^19^, and mental disorders^14^ ^,^ ^15^ - all factors associated with worse health outcomes.

The increase in abusive alcohol consumption among the population with higher level of education may be related to changes in lifestyle, greater access to social environments in which consumption is habitual, and less social disapproval associated with alcohol use. There is also greater cultural tolerance to consumption among the most educated. Although authors of studies indicate a higher prevalence of use in this group^25^ ^,^ ^26^, the adverse effects of consumption are more severe among individuals with lower socioeconomic level^27^, which reinforces social inequalities in health outcomes and points to the need for more equitable and targeted public policies.

In addition, we identified an increase in abusive alcohol consumption in most age groups, with the exception of age groups from 18 to 24 years and from 45 to 54 years, with higher prevalence among adults aged 25 to 34 years. Other researchers have also demonstrated changes in the patterns of lifelong consumption, with a decrease trend among adolescents and young adults (up to 30 years of age) and an increase among individuals aged 31 to 64 years^16^ ^,^ ^28^. This behavior may be related to greater financial autonomy, insertion in social environments that stimulate consumption, and the appreciation of leisure activities throughout adult life.

Regarding the results by race/skin color, there was an increase in prevalence among white, mixed-race, and Black people; however, higher prevalence values of abusive alcohol consumption remain among Black and mixed-race individuals, in line with findings from other studies^29^ ^,^ ^30^. Among the possible explanations for these findings, we highlight individual factors, environmental characteristics as well as social and structural aspects. The stress associated with racism, in addition to greater exposure to vulnerable socioeconomic conditions, contributes to the use of alcohol as a strategy to cope with daily adversity or emotional relief. Likewise, the lower supply of health care and inequalities in access to social and economic opportunities reinforce this pattern of consumption^31^ ^,^ ^32^ ^,^ ^33^.

The national and global targets for reducing alcohol consumption by 10% by 2030 are unlikely to be achieved, due to the progressive increase observed over the years - especially among women. To curb this advance, the WHO recommends the adoption of effective public policies such as: increased taxation on alcoholic beverages; imposition of bans or comprehensive restrictions on advertising of these products in the media; limitation of physical availability by reducing the time for alcohol purchase; strict enforcement of laws prohibiting driving under the influence of alcohol, including the definition of legal limits of alcohol concentration in blood and surveillance actions; and restriction or prohibition of promotions and sponsorship especially aimed at young people^34^.

In Brazil, the implementation of these measures is still limited. Among the few actions in force, the prohibition of driving under the influence of alcohol is highlighted, according to the New Prohibition Law (from Portuguese, *Nova Lei Seca*, Law No. 12.760/2012), which has contributed to the reduction of consumption among drivers^10^. Another recent advance was the inclusion of alcoholic beverages among products subject to selective taxation (taxes on items harmful to health), provided for in the tax reform approved in 2023^35^.

Therefore, it is essential to advance toward more effective regulatory measures, especially through the regulation of the complementary law dealing with the increase in taxes on products harmful to health^36^. It is necessary to strengthen the restriction of access to alcoholic beverages, increase the prohibition of advertising, promotion, and sponsorship of these products as well as to intensify the monitoring of the actions already implemented^34^. Furthermore, Brazilian legislation presents important gaps: currently, only the advertisement of drinks with alcoholic content of more than 13 degrees Gay-Lussac is prohibited, which allows for the wide dissemination of beer advertisements, the most consumed alcoholic beverage in the country^36^. This highlights the urgency of improving the regulatory framework, including regulation of beer advertising, in order to ensure greater effectiveness in consumption control policies.

It should be noted that, although regulatory frameworks are fundamental for controlling alcohol consumption, its isolated application has proved insufficient to address the complexity of the issue. We must acknowledge that alcohol consumption is rooted in cultural, social, and economic practices, thus requiring more integrated and multidimensional approaches. The incorporation of educational, community, and intersectoral strategies - allied with regulation - can increase the scope and effectiveness of public policies.

Among the study limitations, we highlight the cross-sectional design, as telephone interviews with adults who have landlines may not represent the entire population. Nevertheless, this issue is undermined by the use of data weighting factors. The indicator used considers excessive consumption, which measures the consumption of alcoholic beverages on a single occasion in an abusive manner, frequent among young people and individuals with higher level of education, at times of celebration, not necessarily referring to chronic consumption or dependence, which is generally more common among the poorest^25^.

Our results show a worrisome trend of increasing alcohol abuse in the Brazilian population, with emphasis on the increase among women, adults in intermediate age groups, and individuals with higher level of education. Regional and inter-capital variations reinforce the importance of considering territorial and sociodemographic inequalities in the planning of coping actions.

Considering the identified scenario, if current trends are maintained, national and global targets for reducing alcohol consumption by 10% by 2030 will hardly be met. With these findings, we emphasize the urgency of strengthening comprehensive public policies that combine regulatory measures, educational strategies, intersectoral actions, and approaches sensitive to the social and cultural context of consumption. Only through integrated, sustained, and evidence-based responses will it be possible to reverse this trend and promote healthier and equitable environments for the population.

## References

[1] World Health Organization (2024). Alcohol.

[2] Institute for Health Metrics and Evaluation GBD Compare.

[3] GBD 2016 Alcohol Collaborators (2018). Alcohol use and burden for 195 countries and territories, 1990-2016: a systematic analysis for the Global Burden of Disease Study 2016. The Lancet.

[4] Malta DC, da Silva AG, Prates EJS, Alve FTA, Cristo EB, Machado ÍE (2021). Convergence in alcohol abuse in brazilian capitals between genders, 2006 to 2019: What population surveys show. Rev Bras Epidemiol.

[5] Munhoz TN, Santos IS, Nunes BP, Mola CL de, Silva ICM da, Matijasevich A (2017). Tendências de consumo abusivo de álcool nas capitais brasileiras entre os anos de 2006 a 2013: análise das informações do VIGITEL. Cad Saúde Pública.

[6] Petticrew M, Shemilt I, Lorenc T, Marteau TM, Melendez-Torres GJ, O’Mara-Eves A (2017). Alcohol advertising and public health: systems perspectives versus narrow perspectives. J Epidemiol Community Health.

[7] Kindy K, Kreating D (2016). For women, heavy drinking has been normalized. That’s dangerous. The Washington Post.

[8] Gilmore AB, Fabbri A, Baum F, Bertscher A, Bondy K, Chang HJ (2023). Defining and conceptualising the commercial determinants of health. The Lancet.

[9] Nações Unidas no Brasil Objetivos de Desenvolvimento Sustentável.

[10] Malta DC, Bernal RTI, da Silva AG, Lima CM de, Machado ÍE, da Silva MMA (2020). Temporal trend in the prevalence of indicators related to driving a motor vehicle after alcohol consumption, between 2007 and 2018. Rev Bras Epidemiol.

[11] White A, Castle IP, Chen CM, Shirley M, Roach D, Hingson R (2015). Converging patterns of alcohol use and related outcomes among females and males in the United States, 2002 to 2012. Alcohol Clin Exp Res.

[12] Bernal RTI, Iser BPM, Malta DC, Claro RM (2017). Sistema de Vigilância de Fatores de Risco e Proteção para Doenças Crônicas por Inquérito Telefônico (Vigitel): mudança na metodologia de ponderação. Epidemiol Serv Saúde.

[13] Brasil (2021). VIGITEL Brasil 2020: Vigilância de fatores de risco e proteção para doenças crônicas por inquérito telefônico.

[14] Grucza RA, Sher KJ, Kerr WC, Krauss MJ, Lui CK, McDowell YE (2018). Trends in adult alcohol use and binge drinking in the early 21st-century United States: a meta-analysis of 6 national survey series. Alcohol Clin Exp Res.

[15] Grant BF, Chou SP, Saha TD, Pickering RP, Kerridge BT, Ruan WJ (2017). Prevalence of 12-month alcohol use, high-risk drinking, and DSM-IV alcohol use disorder in the United States, 2001-2002 to 2012-2013: Results from the National Epidemiologic Survey on Alcohol and Related Conditions. JAMA Psychiatry.

[16] Keyes KM, Jager J, Mal-Sarkar T, Patrick ME, Rutherford C, Hasin D (2019). Is there a recent epidemic of women’s drinking? A critical review of national studies. Alcohol Clin Exp Res.

[17] Polcin DL, Korcha R, Kerr W, Bond J, Greenfield T (2014). Gender and social pressure to change drinking behavior: Results from the National Alcohol Surveys from 1984 to 2010. Addict Res Theory.

[18] Miech RA, Johnston LD, Patrick ME, O’Malley PM (2025). Monitoring the Future national survey results on drug use, 1975-2024: Overview and detailed results for secondary school students.

[19] Keyes KM, Miech R (2013). Age, period, and cohort effects in heavy episodic drinking in the US from 1985 to 2009. Drug Alcohol Depend.

[20] Almeida-Filho N, Lessa I, Magalhães L, Araújo MJ, Aquino E, Kawachi I (2004). Alcohol drinking patterns by gender, ethnicity, and social class in Bahia, Brazil. Rev Saúde Pública.

[21] Lindsay JM, Supski SD, Lindsay J, Supski S (2017). Youth drinking cultures in a digital world: alcohol, social media and cultures of intoxication.

[22] Salerno PRVO, Briones-Valdivieso C, Motairek I, Dallan LAP, Rajagopalan S, Deo SV (2023). The cardiovascular disease burden attributable to particulate matter pollution in South America: analysis of the 1990-2019 global burden of disease. Public Health.

[23] Nanda S (2005). The essential guide to doing research. Soc Change.

[24] Bagnardi V, Rota M, Botteri E, Tramacere I, Islami F, Fedirko V (2013). Light alcohol drinking and cancer: a meta-analysis. Ann Oncol.

[25] Wendt A, Costa CS, Costa FS, Malta DC, Crochemore-Silva I (2021). Time trend in inequalities in smoking and abusive alcohol consumption in Brazil’s state capitals. Cad Saúde Pública.

[26] Grittner U, Kuntsche S, Gmel G, Bloomfield K (2013). Alcohol consumption and social inequality at the individual and country levels-results from an international study. Eur J Public Health.

[27] Frone MR (2016). The great recession and employee alcohol use: A U.S. population study. Psychol Addict Behav.

[28] White AM, Slater ME, Ng G, Hingson R, Breslow R (2018). Trends in alcohol-related emergency department visits in the United States: results from the nationwide emergency department sample, 2006 to 2014. Alcohol Clin Exp Res.

[29] Garcia GAF, SIlva EKPD, Giatti L, Barreto SM (2021). The intersection race/skin color and gender, smoking and excessive alcohol consumption: cross sectional analysis of the Brazilian National Health Survey, 2013. Cad Saude Publica.

[30] Ribeiro LS, Damacena GN, Szwarcwald CL (2021). Prevalência e fatores sociodemográficos associados ao beber pesado no Brasil: análises transversais da Pesquisa Nacional de Saúde. Rev Bras Epidemiol.

[31] Silva NA, Oliveira JL, Souza J (2016). Alcohol and tobacco consumption among seamstresses from the city of Formiga-Minas Gerais. SMAD.

[32] Chartier K, Caetano R (2010). Ethnicity and health disparities in alcohol research. Alcohol Res Health.

[33] Borrell LN, Kiefe CI, Diez-Roux AV, Williams DR, Gordon-Larsen P (2013). Racial discrimination, racial/ethnic segregation, and health behaviors in the CARDIA study. Ethn Health.

[34] World Health Organization (2024). Tackling NCDs. Best buys and other recommended interventions for the prevention and control of noncommunicable diseases.

[35] Martello A, Carregosa L, Resende T (2024). Governo propõe que “imposto do pecado” seja cobrado sobre cigarros, bebidas alcoólicas, açucaradas, carros e petróleo. G1.

[36] Brasil (2024). Projeto de Lei Complementar nº 68, de 2024.

