# Supplementary material for: Temporal trends in abusive alcohol consumption and their projections for 2030 in Brazilian capitals
Source: Rev Bras Epidemiol. 2026 Jan 12;28:e250057. doi: 10.1590/1980-549720250057 (PMC12799197; doi:10.1590/1980-549720250057)
Supplement: Translation

# Translation: Tendência temporal do consumo abusivo de bebidas alcoólicas e suas projeções para 2030 nas capitais brasileiras

**DOI:** 10.1590/1980-549720250057.2

**Authors:** Deborah Carvalho Malta, Paula Carvalho de Freitas, Crizian Saar Gomes, Guilherme Augusto Veloso, Regina Tomie Ivata Bernal, Alanna Gomes da Silva, Filipe Malta Santos, Sther Luna Abras dos Santos, Paulo Ferrinho

**Affiliations:** I Universidade Federal de Minas Gerais, Escola de Enfermagem - Belo Horizonte (MG), Brasil.; II Ministério da Saúde, Secretaria de Vigilância em Saúde e Ambiente - Brasília (DF), Brasil.; III Universidade Federal de Minas Gerais, Faculdade de Medicina - Belo Horizonte (MG), Brasil.; IV Universidade Federal Fluminense - Niterói (RJ), Brasil.; V Faculdade de Ciências Médicas de Minas Gerais - Belo Horizonte (MG), Brasil.; VI Universidade Nova de Lisboa, Instituto de Higiene e Medicina Tropical - Lisboa, Portugal.

**Keywords:** Consumo excessivo de bebidas alcoólicas, Desenvolvimento sustentável, Inquéritos epidemiológicos, Fatores de tempo, Doença crônica

## Abstract

**Objetivos:**

Analisar a tendência temporal das prevalências do consumo abusivo de bebidas alcoólicas na população adulta brasileira residentes nas capitais brasileiras entre 2006 e 2023 e estimar as projeções para 2030.

**Métodos:**

Estudo de série temporal com dados do sistema de Vigilância de Fatores de Risco e Proteção para Doenças Crônicas por Inquérito Telefônico. Considerou-se consumo abusivo de bebidas alcóolicas a ingestão de cinco ou mais doses para homens e quatro ou mais para mulheres em curto período de tempo. Utilizou-se o modelo de regressão por pontos de inflexão (*joinpoint regression model*) para verificar a existência de tendências. Para as projeções até 2030, foi aplicado o modelo de suavização exponencial.

**Resultados:**

Observou-se aumento na tendência do consumo abusivo de bebidas alcoólicas na população total (de 15,6% em 2006 para 20,8% em 2023) entre as mulheres, na maioria das faixas etárias - exceto entre os grupos de 18 a 24 anos e de 45 a 54 anos -, entre pessoas com 12 anos ou mais de estudo e entre os indivíduos das raças/cores branca, preta e parda. Entre as mulheres, ocorreu aumento em 23 capitais. Considerando as tendências entre 2015 e 2023, as metas de redução 10% até 2030 não deverão ser alcançadas.

**Conclusão:**

O aumento do consumo de álcool na população total e especial entre mulheres e o não alcance da meta de redução mostram a necessidade de avançar nas políticas e programas para deter o consumo de bebidas alcóolicas.

## INTRODUÇÃO

O álcool é uma substância tóxica e psicoativa que pode gerar dependência entre seus consumidores. Em muitas das sociedades, as bebidas alcoólicas são uma parte frequente da convivência social, sendo fácil ignorar ou desconsiderar os danos sociais e de saúde causados pelo seu consumo^1^. Segundo a Organização Mundial da Saúde (OMS), o consumo de bebidas alcoólicas contribui para 2,6 milhões de mortes todos os anos em todo o mundo, sendo 1,6 milhão causada por doenças crônicas não transmissíveis (DCNT), 700 mil mortes por lesões e 300 mil mortes por doenças transmissíveis^1^.

O uso nocivo de bebidas alcoólicas é responsável por 4,7% da carga global de doenças, sendo 6,9 e 2% entre homens e mulheres, respectivamente. O álcool é o principal fator de risco para mortalidade prematura e incapacidade entre as pessoas com 20 a 39 anos, sendo responsável por 13% de todas as mortes nessa faixa etária. As populações desfavorecidas e especialmente vulneráveis apresentam taxas mais elevadas de morte e hospitalização relacionadas ao consumo de bebidas alcóolicas^1^.

No Brasil, o consumo de bebidas alcoólicas foi o sétimo fator de risco em 2021 para perda de anos de vida ajustados por incapacidade (*disability-adjusted life years -* DALYs), acarretando 2.710.367 milhões (3,45%) de DALYs^2^.

Entre os indivíduos que consumiram bebidas alcóolicas de forma abusiva em 2020, 59,1% tinham entre 15 e 39 anos e 76,9% eram do sexo masculino^3^. Mesmo as prevalências de consumo sendo mais elevadas entre homens, têm ocorrido mudanças nesse padrão, e as mulheres têm aumentado seu consumo, tendendo à convergência entre as prevalências por sexo^4^. Essa mudança pode estar relacionada ao crescimento do *marketing* destinado às mulheres por parte da indústria do álcool, visando ampliar sua base de consumo, fenômeno que tem sido descrito como determinante comercial em saúde, ou a pressão da indústria para mudar padrões de consumo locais e ampliar sua lucratividade^4^ ^,^ ^5^ ^,^ ^6^ ^,^ ^7^.

A indústria age em diversas frentes e estratégias, entre elas no ambiente cultural, por meio de propagandas e atuação nas mídias sociais, buscando engajamentos, disseminando *fake news*, visando estabelecer novos parâmetros de comportamento. Certos países e regiões, como países de baixa e média renda, enfrentam maior pressão de atores transnacionais^8^.

No intuito de reduzir o consumo abusivo de bebidas alcoólicas, diversas iniciativas globais e nacionais foram adotadas. Em maio de 2010, a Assembleia Mundial da Saúde aprovou a Estratégia Global para Reduzir o Uso Nocivo do Álcool^1^; em 2018, foi lançada a iniciativa SAFER pela OMS, e no Brasil em 2019^1^ ^,^ ^2^. Essas iniciativas têm como objetivo apoiar os países na implementação de intervenções de alto impacto e com boa relação de custo-eficácia que comprovadamente reduzem os danos causados pelo consumo de bebidas alcóolicas^1^.

O monitoramento do consumo de bebidas alcoólicas compõe as metas da Agenda do Desenvolvimento Sustentável^9^, que prevê a redução do consumo de bebidas alcoólicas em 10% até 2030. No Brasil, o Plano de Ações Estratégicas para o Enfrentamento das Doenças Crônicas e Agravos Não Transmissíveis também propõe metas semelhantes, entretanto, no Brasil, não tem sido observada redução^10^. Portanto, monitorar o consumo de bebidas alcoólicas a fim de identificar os grupos com maior exposição pode contribuir para a elaboração de políticas e programas de saúde de enfrentamento ao problema.

Nesse sentido, este estudo teve como objetivos: analisar a tendência temporal das prevalências do consumo abusivo de bebidas alcoólicas na população adulta brasileira residente nas capitais brasileiras entre 2006 e 2023 e estimar as projeções para 2030.

## MÉTODOS

### Desenho do estudo

Estudo de série temporal utilizando dados do sistema de Vigilância de Fatores de Risco e Proteção para Doenças Crônicas por Inquérito Telefônico (Vigitel), entre os anos de 2006 e 2023.

### Contexto

Vigitel é um inquérito telefônico de base populacional realizado anualmente pelo Ministério da Saúde que coleta informações sobre as DCNT, bem como sobre os principais fatores de risco e de proteção relacionados a essas doenças na população adulta brasileira (≥18 anos). Até o ano de 2021, os procedimentos de amostragem visavam obter, em cada uma das capitais dos 26 estados brasileiros e no Distrito Federal, amostras probabilísticas da população de adultos que residiam em domicílios servidos por, ao menos, uma linha telefônica fixa. Em 2023, foi incorporada a modalidade de entrevista por meio de telefone celular. Nas edições realizadas entre 2006 e 2019, estabeleceu-se um tamanho amostral de no mínimo dois mil adultos em cada cidade. No entanto, nos anos de 2020 e 2021, por causa das dificuldades impostas pela pandemia de COVID-19 à coleta dos dados, reduziu-se o tamanho amostral, para cerca de mil indivíduos em cada cidade. Para o ano de 2023, nova redução foi necessária, fixando-se um mínimo de 800 entrevistas em cada uma das localidades. Ademais, a rápida deterioração da cobertura de telefonia fixa no país motivou que metade das entrevistas fosse realizada por telefone móvel a fim de permitir a estimação de dados de boa qualidade (com amostra final de 400 entrevistas por telefone fixo e 400 por telefone móvel em cada localidade)^11^ ^,^ ^12^.

O processo de amostragem do Vigitel ocorre em duas etapas. A primeira etapa consiste no sorteio das linhas telefônicas por cidade. A seguir, as linhas sorteadas em cada cidade são sorteadas mais uma vez e divididas em réplicas (de 200 linhas para telefones fixos e 500 para os móveis). A segunda etapa (somente para os casos de telefone fixo) consiste no sorteio de um dos adultos (≥18 anos de idade) para a entrevista^13^.

Uma vez que a amostra de adultos entrevistados pelo Vigitel foi extraída do cadastro das linhas telefônicas (fixos residenciais e móveis), permitindo inferências populacionais somente para a população adulta com telefone em cada uma das localidades, pesos (denominados pesos pós-estratificação) são atribuídos a cada indivíduo a fim de igualar a composição sociodemográfica estimada para a amostra Vigitel em cada cidade à composição sociodemográfica que se estima para a população adulta total da mesma cidade^12^. O peso pós-estratificação de cada indivíduo da amostra Vigitel foi calculado pelo método *rake* ^*12*^ ^,^ ^13^.

Mais informações sobre o processo de amostragem e seleção podem ser encontradas nos relatórios anuais de divulgação dos resultados^13^.

### Variáveis

O presente estudo analisou o consumo abusivo de bebida alcoólica, segundo as seguintes perguntas:

• Para homens: nos últimos 30 dias, o senhor chegou a consumir cinco ou mais doses de bebida alcoólica em uma única ocasião?;
• Para mulheres: nos últimos 30 dias, a senhora chegou a consumir quatro ou mais doses de bebida alcoólica em uma única ocasião?

O indicador analisado consumo abusivo de bebidas alcoólicas constitui o consumo de um volume excessivo de bebidas alcóolicas em um curto espaço de tempo, sendo cinco ou mais doses para homens e quatro ou mais doses para as mulheres^2^ ^,^ ^13^.

### Análise dos dados

A prevalência de consumo abusivo de bebidas alcoólicas foi calculada para população total e estratificada segundo sexo (feminino e masculino); faixa etária (18 a 24; 25 a 34; 35 a 44; 45 a 54; 55 a 64; e 65 anos ou mais); escolaridade (0 a 8; 9 a 11; e 12 anos ou mais de estudo); raça/cor (branca, preta, amarela, parda e indígena); regiões (Norte, Nordeste, Centro-Oeste, Sudeste e Sul); e capitais brasileiras.

A análise das tendências temporais do consumo abusivo de álcool foi realizada por meio do modelo de regressão por pontos de inflexão (*joinpoint regression model*), com o objetivo de identificar mudanças no comportamento dos dados ao longo do período de 2006 a 2023. Para a avaliação da tendência durante o período, utilizou-se a variação percentual anual média (*average annual percent change* - AAPC). Considerou-se a existência de tendência significativa quando a AAPC foi estatisticamente diferente de 0, ou seja, quando o valor de *p* do teste t de Student foi igual ou inferior a 5%, rejeitando-se a hipótese nula de ausência de tendência. A direção da tendência foi interpretada com base no sinal da AAPC: valores positivos indicam crescimento, enquanto valores negativos sugerem redução.

Em função das metas pactuadas dos Objetivos de Desenvolvimento Sustentável de redução de 10% do consumo de bebidas alcóolicas entre 2015 e 2030, foi realizada a projeção para 2030 para população total e segundo sexo, usando o modelo de suavização exponencial aplicado em todos os dados observados.

Todas as análises consideraram os fatores de ponderação, levando-se em conta a probabilidade desigual que os indivíduos residentes em domicílios com maior número de linhas telefônicas ou menor número de moradores tiveram em participar da amostra.

Para análise dos dados, utilizaram-se os *softwares* RStudio e Joinpoint.

### Aspectos éticos

O consentimento livre e esclarecido foi obtido verbalmente durante o contato telefônico com os entrevistados. O Vigitel foi aprovado pela Comissão Nacional de Ética em Pesquisa com Seres Humanos do Ministério da Saúde (CAAE: 65610017.1.0000.0008), parecer número 4.324.071.

## Declaração de disponibilidade de dados:

Os dados usados neste estudo são públicos e podem ser acessados em: https://svs.aids.gov.br/download/Vigitel/.

## RESULTADOS

Entre 2006 e 2023, foi entrevistado um total de 806.169 indivíduos, distribuídos da seguinte forma por ano: 2006 (54.369), 2007 (54.251), 2008 (54.353), 2009 (54.367), 2010 (54.339), 2011 (54.144), 2012 (45.448), 2013 (52.929), 2014 (40.853), 2015 (54.174), 2016 (53.210), 2017 (53.034), 2018 (52.395), 2019 (52.443), 2020 (27.077), 2021 (27.093) e 2023 (21.690).

Observou-se aumento nas prevalências de consumo abusivo de bebidas alcoólicas entre a população residente nas capitais brasileiras, passando de 15,7% em 2006 para 20,8% em 2023. Ao analisar as tendências por sexo, notaram-se aumento significativo entre as mulheres - de 7,8% em 2006 para 15,2% em 2023 - e estabilidade entre os homens, com prevalência de 25% em 2006 e 27,3% em 2023. O consumo abusivo de bebidas alcoólicas também aumentou em todas as faixas etárias, exceto entre os jovens de 18 a 24 anos e os adultos de 45 a 54. Entre pessoas com 12 anos ou mais de estudo, a prevalência subiu de 18,1 para 24%. Observaram-se crescimento também nas raças/cores branca (14,7 para 21%), preta (18,8 para 23,2%) e parda (16,1 para 20,3%) e redução na amarela (16,1 para 14,3%). Regionalmente, houve aumento nas prevalências nas regiões Centro-Oeste (15,5 para 23,6%), Nordeste (18,4 para 21,4%), Sudeste (14,3 para 20,8%) e Sul (13,2 para 20,7%) (Tabela 1).

**Tabela 1.** Tendência temporal das prevalências do consumo abusivo de bebidas alcóolicas segundo sexo, faixa etária, escolaridade, raça. Vigilância de Fatores de Risco e Proteção para Doenças Crônicas por Inquérito Telefônico (Vigitel), 2006 a 2023.

	2006	2007	2008	2009	2010	2011	2012	2013	2014	2015	2016	2017	2018	2019	2020	2021	2023	AAPC
Total	15,7	16,5	17,2	18,4	18,1	16,5	18,4	16,4	16,5	17,2	19,1	19,1	17,9	18,8	20,9	18,3	20,8	1,1*
Sexo																		
Masculino	25,0	25,5	26,1	28,3	27,0	25,3	27,9	24,2	24,8	25,3	27,3	27,1	26,0	25,3	26,6	25,0	27,3	0,0
Feminino	7,8	8,7	9,6	10,0	10,5	9,0	10,3	9,7	9,4	10,2	12,1	12,2	11,0	13,3	16,0	12,7	15,2	3,3*
Escolaridade (anos)																		
0 a 8	13,6	13,9	14,5	14,5	14,0	13,4	15,0	12,8	12,3	13,2	14,2	13,8	13,0	12,4	15,0	11,7	14,4	-0,5
9 a 11	17,0	18,5	19,2	19,8	19,6	17,5	19,4	17,5	18,4	18,1	19,2	20,2	19,1	20,0	22,5	19,4	22,1	0,7
≥12	18,1	18,7	19,5	23,7	22,9	20,0	22,0	19,7	19,5	20,9	24,0	22,8	21,2	23,1	23,8	22,5	24,0	1,1*
Faixa etária (anos)																		
18 a 24	19,0	22,3	21,3	23,3	22,0	20,2	21,8	19,0	18,2	20,2	22,1	23,8	23,0	25,8	25,0	19,3	21,4	0,7
25 a 34	21,7	21,6	22,1	23,9	24,1	21,3	24,7	22,7	23,2	23,5	25,8	27,7	24,2	26,3	30,9	25,5	29,8	1,6*
35 a 44	17,6	16,5	19,3	20,0	19,8	18,2	20,0	17,5	18,0	19,4	21,2	22,2	21,7	20,9	21,5	20,0	24,7	1,5*
45 a 54	13,2	14,3	15,2	16,8	15,9	14,8	16,6	15,0	15,1	15,5	18,2	15,8	14,7	15,8	18,2	17,9	21,1	1,3
55 a 64	6,6	9,6	10,3	10,4	10,7	10,6	11,9	10,5	11,0	11,0	12,6	10,3	11,0	11,2	13,8	13,2	11,4	3,3*
≥65	2,5	2,7	3,3	4,1	4,4	4,5	5,0	4,0	3,8	3,7	4,6	3,0	4,1	4,1	5,7	5,8	5,4	5,8*
Raça/cor																		
Branca	14,7	15,2	15,8	18,1	17,4	15,4	17,8	15,8	15,7	16,6	18,1	18,9	16,5	18,0	20,7	16,9	21,0	1,39*
Preta	18,8	21,5	21,7	23	21,1	20,6	21,7	19,5	20,6	20,3	25	22	21,7	24	25,5	24	23,2	1*
Amarela	16,1	17,0	17,8	18,2	18,3	14,2	16,4	17,0	17,3	16,6	12,7	10,6	14,0	14,2	11,4	10,0	14,3	-2,6*
Parda	16,1	15,1	8,8	12,3	5,3	16,8	18,5	17,2	17,0	17,6	20,0	19,6	19,1	19,0	21,0	18,5	20,3	4*
Indígena	8,7	44,1	13,2	17,8	21,9	20,8	26,1	16,3	12,5	23,9	20,0	20,7	15,0	18,1	20,0	14,4	23,8	0,3
Região																		
Centro-Oeste	15,5	16,7	17,3	18,6	18,7	15,0	19,3	17,1	18,5	20,8	22,0	23,7	19,7	21,8	24,3	21,1	23,6	2,4*
Nordeste	18,4	19,2	19,9	20,8	20,8	19,2	20,5	18,0	16,7	17,2	20,1	19,4	18,7	19,0	20,4	18,8	21,4	0,8*
Norte	17,2	17,3	19,7	18,4	18,3	16,0	16,6	14,5	14,8	15,1	17,0	16,5	16,7	16,7	15,7	16,5	16,1	0,1
Sudeste	14,3	15,2	16,0	17,6	16,9	15,9	18,1	16,2	16,5	17,2	18,7	18,5	17,7	18,8	21,8	18,3	20,8	1,7*
Sul	13,2	14,5	12,8	15,4	15,3	14,1	15,2	13,4	15,4	14,1	16,3	17,7	15,7	16,6	19,5	15,1	20,7	1,8*

AAPC: *average annual percent change* (variação percentual anual média); *valor-p significativo (<0,05).

As Tabela 2 e 3 apresentam a tendência do consumo de bebidas alcóolicas nas capitais estratificada por sexo. Entre as mulheres, em quase todas as capitais houve tendência de aumento, exceto Belém (PA), Macapá (AP), Manaus (AM) e Maceió (AL), que permaneceram estáveis (Tabela 2). Ao contrário, entre os homens, ocorreram redução de consumo em cinco capitais - Belém, Manaus, Natal (RN), Recife (PE), São Luís (MA) - e aumento em apenas duas capitais - Distrito Federal e São Paulo (SP) (Tabelas 3).

**Tabela 2.** Tendência temporal das prevalências do consumo abusivo de bebidas alcóolicas entre o sexo feminino, nas regiões e capitais brasileiras. Vigilância de Fatores de Risco e Proteção para Doenças Crônicas por Inquérito Telefônico (Vigitel), 2006 a 2023.

	Ano																	AAPC
	2006	2007	2008	2009	2010	2011	2012	2013	2014	2015	2016	2017	2018	2019	2020	2021	2023	
Local																		
Norte	7,7	7,4	9,2	8,6	8,1	7,9	8,2	7,0	6,6	6,5	8,5	9,7	9,2	9,4	9,6	10,9	10,3	1,8*
Belém	8,6	7,6	9,8	10,6	9,0	10,3	11,4	8,0	8,5	5,9	8,5	11,4	9,0	9,9	9,1	9,8	10,1	0,4
Boa Vista	6,4	8,7	8,5	9,6	8,8	9,3	7,8	7,2	6,1	8,3	11,9	11,1	10,0	9,5	12,8	12,8	13,5	3,2*
Macapá	8,4	9,0	5,3	9,4	9,5	8,6	7,7	8,7	7,1	8,1	9,2	8,6	10,5	6,9	9,5	13,7	9,9	1,7
Manaus	6,8	6,5	9,5	6,0	5,9	5,1	5,1	4,6	4,5	5,2	7,7	8,0	8,1	8,7	8,2	9,8	8,4	0,8
Palmas	9,9	8,9	9,6	10,1	12,1	9,7	11,0	10,7	8,9	11,1	13,1	11,8	12,9	17,4	12,4	13,0	13,6	2,6*
Porto Velho	7,3	7,3	10,0	10,9	10,1	9,0	10,7	10,6	9,0	8,0	7,5	10,6	8,9	9,3	12,4	14,4	13,0	4,4*
Rio Branco	7,3	7,3	8,0	7,7	8,5	7,0	6,3	6,3	5,5	8,0	7,2	8,6	11,2	7,7	10,9	10,2	12,1	2,7*
Nordeste	9,0	10,2	10,5	10,7	11,9	10,9	11,0	10,1	8,7	9,7	12,4	11,6	11,7	12,9	14,5	13,2	14,6	3,1*
Aracaju	8,2	7,6	9,3	8,3	11,9	9,2	9,3	11,5	11,0	9,1	13,6	13,2	11,4	12,7	15,4	11,2	16,8	3,7*
Fortaleza	7,5	9,2	6,8	8,2	8,0	7,5	6,5	7,3	6,6	7,3	8,0	9,9	9,0	12,2	9,9	13,7	9,6	2,5*
João Pessoa	5,8	9,0	8,2	8,7	10,6	8,4	7,4	6,4	5,1	5,2	7,3	9,7	7,4	9,3	10,1	13,0	11,1	4,3*
Maceió	8,0	9,0	8,3	11,6	9,9	9,8	9,8	7,1	6,8	7,4	14,4	9,2	8,8	8,3	12,5	8,4	11,9	0,9
Natal	5,6	7,7	9,0	7,8	8,0	7,3	9,3	6,9	8,4	6,7	10,4	9,1	8,0	8,6	14,4	10,3	10,9	2,8*
Recife	11,9	10,3	12,0	9,3	13,5	11,9	13,2	10,3	10,8	10,3	12,9	12,1	14,0	13,5	16,9	15,2	16,6	2,6*
Salvador	11,5	13,4	15,2	15,3	16,9	16,0	16,7	13,9	11,5	15,4	18,3	15,5	16,7	18,1	21,6	15,2	21,9	2,2*
São Luiz	6,7	9,4	9,4	10,1	10,6	10,6	9,4	11,9	6,4	6,9	9,9	10,8	10,5	11,3	10,6	13,7	13,0	2,2*
Teresina	9,0	9,9	9,5	10,0	11,9	9,9	10,6	12,0	8,9	10,5	12,3	9,8	11,0	11,5	11,3	10,3	12,8	1,1*
Centro-Oeste	8,3	8,7	9,1	12,8	11,2	8,6	11,5	10,2	10,4	13,6	14,4	14,3	11,2	15,2	18,8	14,8	17,0	4,1*
Distrito Federal	8,7	8,5	10,1	14,9	12,6	8,7	13,1	9,3	11,5	15,4	15,1	16,3	11,7	17,1	18,6	16,3	20,5	4,5*
Campo Grande	8,6	8,4	8,0	10,7	10,6	7,0	9,4	12,6	7,0	7,7	11,7	10,0	9,4	13,3	19,7	15,4	14,8	3,8*
Cuiabá	7,7	9,1	9,7	9,8	10,8	10,4	11,9	11,9	11,2	14,1	14,5	14,3	12,6	10,9	16,4	12,3	16,6	3,5*
Goiânia	7,4	9,3	7,4	11,3	9,2	8,8	9,5	9,9	9,8	13,2	14,5	12,9	10,8	14,4	19,7	12,5	11,7	3,8*
Sudeste	7,2	8,5	9,7	9,6	10,4	8,5	10,5	10,4	10,4	10,7	12,3	13,0	11,1	14,2	17,6	12,7	15,7	4,0*
Belo Horizonte	12,1	10,3	13,1	14,1	12,8	12,1	13,0	14,5	16,0	11,9	15,3	16,5	16,4	15,2	15,3	16,0	18,0	2,3*
Rio de Janeiro	9,6	11,8	12,1	13,3	12,6	12,5	13,3	11,0	12,2	14,4	13,7	13,2	12,7	17,6	20,4	16,6	17,7	3,0*
São Paulo	4,7	6,1	7,5	6,3	8,4	5,3	8,3	9,2	8,1	8,2	10,7	12,1	9,0	12,1	16,7	9,7	14,0	5,7*
Vitória	10,8	10,0	10,8	13,9	12,9	12,4	12,3	11,3	10,6	14,2	14,9	13,4	15,5	12,3	14,0	15,3	18,0	2,4*
Sul	5,8	7,5	7,1	8,0	8,3	7,7	8,2	6,8	7,8	8,6	10,8	9,8	10,5	11,1	15,4	10,5	17,4	4,9*
Curitiba	7,7	9,1	9,7	9,8	10,8	10,4	11,9	11,9	11,2	14,1	14,5	14,3	12,6	10,9	16,4	12,3	16,6	3,5*
Florianópolis	7,1	9,3	9,9	10,0	12,6	8,6	9,9	10,9	11,4	12,1	10,6	12,5	14,2	13,5	20,5	17,6	18,8	4,9*
Porto Alegre	6,7	9,4	8,8	9,2	10,2	8,6	10,5	8,7	10,2	10,0	12,5	7,3	12,6	13,0	18,0	10,5	20,7	4,2*

AAPC: *average annual percent change* (variação percentual anual média); *valor-p significativo (<0,05).

**Tabela 3.** Tendência temporal das prevalências do consumo abusivo de bebidas alcóolicas entre o sexo masculino, nas regiões e capitais brasileiras. Vigilância de Fatores de Risco e Proteção para Doenças Crônicas por Inquérito Telefônico (Vigitel), 2006 a 2023.

Local	Ano																	AAPC
	2006	2007	2008	2009	2010	2011	2012	2013	2014	2015	2016	2017	2018	2019	2020	2021	2023	
Norte	27,6	28,1	31,1	29,1	29,5	24,7	25,8	22,8	23,6	24,5	26,4	23,9	24,9	24,6	22,4	22,7	22,4	-2,6*
Belém	26,9	32,5	37,0	32,8	32	24,4	27,2	27,8	25,9	24,2	29,9	26,8	27,5	25,4	25,1	21,9	26,5	-1,6*
Boa Vista	23,9	29,4	25,4	31,1	25,5	21,8	26,9	27,7	21,1	21,6	24,5	24	30,2	28,4	25,7	23,4	28,1	0,0
Macapá	29,3	31	34,2	37,4	29,2	25,9	30,7	27,8	27,6	25,1	30,2	23,7	30,2	26	25,1	27,6	26,8	-1,3
Manaus	28,6	24,6	28,6	25,9	29,7	25	22,8	17,2	20,9	24,7	23,2	19,9	19,9	21,9	17,6	20,2	17,1	-2,6*
Palmas	28,9	30,1	31,3	27,1	31,9	29,3	32,1	29,1	25,7	27,6	34,9	33,2	32,5	28,5	34,7	32,5	26,6	0,3
Porto Velho	28	26	28,2	27,5	25,7	26,4	29,1	21,2	23,3	28,8	24,6	27,2	28,2	24,5	21,3	28,4	24,7	-0,5
Rio Branco	25,1	23,3	22,1	21,3	23,9	20,2	20,2	18,9	25,4	18,4	22,4	21,8	20,2	28,6	23,5	17,4	18,3	-0,7
Nordeste	29,8	30,1	31,3	33	31,7	29,3	31,9	27,5	26,3	26,2	29,4	28,9	27,2	26,4	27,4	25,7	29,4	-0,9*
Aracaju	29,3	23,7	32	34,1	34,8	29,3	31,3	34,7	23,5	26,4	31,8	31,2	26,5	28,4	33,5	24,7	25,7	-0,6
Fortaleza	25,9	27,6	29,9	32,6	26,7	26,8	27,9	21,3	22,4	22,4	28	25,7	21,9	24,1	22,2	23,8	25,1	-1,3
João Pessoa	28,6	31,1	29,6	32,3	28	27,8	29,3	26	22,3	24,7	23,8	29,3	26,4	26,2	25,1	27,9	29,1	-0,6
Maceió	27,2	29,2	28,7	32,1	31,9	28,7	28,4	27,3	27,5	19	28,4	29,2	28,8	23,8	20,6	19	34,2	-1,2
Natal	27,5	31,3	28,9	31	34,3	28,6	29	23,9	24,9	24,3	29,7	28,5	26,1	20,8	25,8	21,4	20,8	-2,1*
Recife	32,5	30,7	28,5	29,1	35,1	28,5	30,7	28,2	22,8	27	28	29,1	28,2	25,6	26,4	24,6	27,1	-1,2*
Salvador	32,9	29,3	34	34,7	32,8	30,2	38,5	30,4	32	31	32,9	30,6	31,6	31,7	34	31,5	37,5	0,2
São Luiz	30	36,5	30,7	34,8	34,4	30,7	30,7	32,6	29,4	27,6	27	31,2	25,6	24,5	29,3	23	24,7	-2,0*
Teresina	31,1	34,4	38,1	36,4	31,2	36,3	34,8	29,7	28	30,2	31,9	27,9	29,1	26,6	28,1	28,5	33	0,2
Centro-Oeste	23,3	26,3	26,6	25,2	27,2	22,3	28	24,9	27,8	29	30,6	34,3	29,3	29,4	30,4	28,3	31,1	1,5*
Distrito Federal	22,1	26,4	25,8	23,3	28,1	21	27,5	24,5	31,9	33,7	31,1	36,4	30,7	30,9	30,8	29,7	31,9	2,1*
Campo Grande	20,6	28,1	25,5	27,4	23,9	19	28,5	23	24,9	21,5	28,2	29,9	30,8	27,4	28,9	24,7	30,2	1,3
Cuiabá	28,9	31,2	29,5	28	31,1	30,4	28,3	31,1	26,5	28,1	34,5	36,2	27,6	33,1	30,9	35	33	0,8
Goiânia	25	22,8	27,3	26,2	25,6	23,2	28,7	24,2	22,1	24,8	29,3	32	26,5	26	30,2	25,2	29,2	0,9
Sudeste	22,5	23,2	23,4	27,1	24,5	24,7	27	23,1	23,6	25	26,3	24,9	25,5	24,3	26,6	25	26,8	0,6
Belo Horizonte	27,1	29,3	30	34,1	30,6	28,1	30,6	25,5	29,5	27,5	29,2	26	29,5	27,3	30,2	36,2	27,7	0,0
Rio de Janeiro	25,5	27,4	27,8	29,6	26	24,6	25,6	26	25,6	28,2	31,5	26,6	26,7	28,4	23,5	23,8	25,8	-0,3
São Paulo	19,6	19,3	19,2	24	22,2	23,9	26,9	20,8	21,2	22,5	22,7	23,6	23,8	21,2	27,6	23	27,1	1,3*
Vitória	25,4	31	28,6	29	28,9	27,5	29,4	26,6	24,6	26	30,3	27,1	30,4	26,4	29,5	32,6	29,2	0,3
Sul	22,1	22,6	19,4	24,1	23,4	21,6	23,4	21,2	24,3	20,5	22,7	27	21,8	23	24,2	20,5	24,5	0,4
Curitiba	20,3	21,6	18,2	22,3	21,2	22,4	21,8	20,5	20,8	19,1	23,3	28,4	22	22,4	21,6	22,7	24,6	0,9
Florianópolis	29,3	27,1	25,5	29,9	29,1	25,6	34	26,4	34,4	27,8	31,1	32,9	31	29,3	33,5	25,8	28,5	0,4
Porto Alegre	22,2	22,4	19,1	24,9	24,4	19,4	22	20,4	25,3	19,9	19,2	23,2	18,3	21,6	24,4	15,6	22,9	-0,6

AAPC: *average annual percent change* (variação percentual anual média); *valor-p significativo (<0,05).

A Figura 1 apresenta as projeções da prevalência do consumo abusivo de bebidas alcoólicas até 2030. A meta de redução em 10%, tendo como linha de base a prevalência de 2015, é atingir 15,5% em 2030 para população total, 22,8% no sexo masculino e 9,2% no sexo feminino. Entretanto, considerando as tendências de 2015 a 2023, a projeção para 2030 é de 22,2 (população total), 26,5 (sexo masculino) e 19,3% (sexo feminino), ou seja, as metas não deverão ser alcançadas.

**Figura 1.** Tendências e projeção da prevalência do consumo abusivo de bebidas alcóolicas na (a) população adulta, (b) feminina e (c) masculina. Vigilância de Fatores de Risco e Proteção para Doenças Crônicas por Inquérito Telefônico (Vigitel), Brasil, 2006-2030.

## DISCUSSÃO

O estudo aponta para uma tendência de aumento do consumo abusivo de bebidas alcoólicas na população total e entre as mulheres, enquanto ela se mantém estável entre os homens. Observou-se crescimento do consumo abusivo nas faixas etárias de 25 a 34 anos, 35 a 44 anos, 55 a 64 anos e 65 anos ou mais, bem como entre a população com 12 anos ou mais de escolaridade e nas raças/cores branca, parda e preta. Houve variações nas tendências segundo as regiões e capitais analisadas. Entre os homens, foram identificados aumento em duas capitais e redução em cinco. Já entre as mulheres, observou-se aumento em 23 capitais. Considerando as tendências entre 2015 e 2023, as metas de redução de 10% no consumo abusivo de álcool até 2030 não deverão ser alcançadas.

O aumento do consumo de bebidas alcoólicas entre as mulheres e a estabilidade entre os homens, evidenciados neste estudo, têm sido relatados em diversos estudos^14^ ^,^ ^15^ ^,^ ^16^ ^,^ ^17^ ^,^ ^18^, apontando igualmente para o estreitamento das diferenças entre os sexos ao longo do tempo^4^ ^,^ ^11^ ^,^ ^18^ ^,^ ^19^. Ademais, observou-se crescimento do consumo abusivo entre as mulheres na maioria das capitais, indicando tendência de aproximação das prevalências entre homens e mulheres no período analisado. Sabe-se que os padrões de consumo de álcool são influenciados por fatores locais, regionais e culturais^20^. Dados da Pesquisa Nacional de Saúde de 2019 mostraram que as maiores prevalências entre mulheres foram registradas na Bahia, Sergipe e Mato Grosso do Sul^20^.

O aumento do consumo entre as mulheres tem sido atribuído, em parte, às estratégias de *marketing* da indústria alcooleira e ao papel das mídias sociais, que passaram a direcionar suas campanhas ao público feminino^6^ ^,^ ^21^. A criação de produtos voltados especificamente às mulheres - como bebidas com sabores adocicados, embalagens coloridas e *drinks* personalizados - tem reforçado esse movimento^22^. A literatura tem discutido amplamente o papel dos determinantes comerciais da saúde na promoção do consumo entre mulheres^8^, destacando a atuação estratégica da indústria na construção de narrativas que associam o consumo ao empoderamento, à liberdade e à inserção social. Além disso, mudanças socioculturais, como o aumento da escolaridade, a maior participação feminina no mercado de trabalho, a adoção de novos estilos de vida^4^, a busca por maior igualdade entre os gêneros e a incorporação de comportamentos tradicionalmente associados ao universo masculino^5^, também podem contribuir para esse cenário.

Estudos apontam que o aumento do uso abusivo entre mulheres tem implicado em diversos efeitos adversos, como riscos durante a gestação e para o bebê^23^, maior incidência de câncer de mama^24^, doenças cardiovasculares^19^ e transtornos mentais^14^ ^,^ ^15^ - todos fatores associados a piores desfechos em saúde.

O aumento do consumo abusivo entre a população de maior escolaridade pode estar relacionado a mudanças nos estilos de vida dessa população, maior acesso a espaços sociais em que o consumo é naturalizado e menor reprovação social associada ao uso de álcool. Observa-se, ainda, maior tolerância cultural ao consumo entre os mais escolarizados. Embora estudos indiquem maior prevalência de uso abusivo nesse grupo^25^ ^,^ ^26^, os efeitos adversos do consumo são mais intensos entre indivíduos com menor nível socioeconômico^27^, o que reforça as desigualdades sociais nos desfechos em saúde e aponta para a necessidade de políticas públicas mais equitativas e direcionadas.

Além disso, o estudo identificou aumento do consumo abusivo de bebidas alcoólicas na maioria das faixas etárias, com exceção dos grupos de 18 a 24 anos e de 45 a 54 anos, com maiores prevalências entre adultos de 25 a 34 anos. Outros estudos também revelaram mudanças nos padrões de consumo ao longo da vida, com tendência de redução entre adolescentes e adultos jovens (até 30 anos) e aumento entre indivíduos de 31 a 64 anos^16^ ^,^ ^28^. Esse comportamento pode estar relacionado à maior autonomia financeira, à inserção em ambientes sociais que estimulam o consumo e à valorização de práticas de lazer e entretenimento durante a vida adulta.

No que se refere aos resultados por raça/cor, verificou-se aumento das prevalências entre pessoas brancas, pardas e pretas, entretanto as maiores prevalências de consumo abusivo de bebidas alcoólicas permanecem entre indivíduos pretos e pardos, em consonância com achados de outros estudos^29^ ^,^ ^30^. Entre as possíveis explicações para esses achados, destacam-se fatores individuais, características ambientais, além de aspectos sociais e estruturais. O estresse associado ao racismo, somado à maior exposição a condições socioeconômicas vulneráveis, contribui para o uso do álcool como estratégia de enfrentamento das adversidades cotidianas ou alívio emocional. Igualmente, a menor oferta de cuidados em saúde e desigualdades no acesso a oportunidades sociais e econômicas reforçam esse padrão de consumo^31^ ^,^ ^32^ ^,^ ^33^.

O estudo afirma que as metas nacionais e globais de redução do consumo de bebidas alcoólicas em 10% até 2030 provavelmente não serão alcançadas, em razão do aumento progressivo observado ao longo dos anos - especialmente entre as mulheres. Para conter esse avanço, a OMS recomenda a adoção de políticas públicas eficazes, como: aumento da tributação sobre bebidas alcoólicas; imposição de proibições ou restrições abrangentes à publicidade desses produtos na mídia; limitação da disponibilidade física por meio da redução do horário; aplicação rigorosa de leis que proíbam a condução de veículos sob efeito de álcool, incluindo a definição de limites legais de concentração alcoólica no sangue e ações de fiscalização; e restrição ou proibição de promoções e patrocínios voltados especialmente ao público jovem^34^.

No Brasil, a implementação dessas medidas ainda é limitada. Entre as poucas ações em vigor, destaca-se a proibição do consumo de álcool por condutores de veículos, conforme a Nova Lei Seca (Lei nº 12.760/2012), que tem contribuído para a redução do consumo entre motoristas^10^. Outro avanço recente foi a inclusão de bebidas alcoólicas entre os produtos sujeitos à tributação seletiva (impostos sobre itens prejudiciais à saúde), prevista na reforma tributária aprovada em 2023^35^.

Portanto, é fundamental avançar em medidas regulatórias mais eficazes, especialmente por meio da regulamentação da lei complementar que trata do aumento de impostos sobre produtos nocivos à saúde^36^. É necessário fortalecer a restrição ao acesso às bebidas alcoólicas, ampliar a proibição da publicidade, promoção e patrocínio desses produtos, bem como intensificar a fiscalização das ações já implementadas^34^. Adicionalmente, a legislação brasileira apresenta lacunas importantes: atualmente, apenas é proibida a propaganda de bebidas com teor alcoólico superior a 13 graus Gay-Lussac, o que permite a ampla veiculação de anúncios de cerveja, a bebida alcoólica mais consumida no país^36^. Isso evidencia a urgência de se aperfeiçoar o marco regulatório, incluindo a regulação da publicidade de cervejas, de forma a garantir maior efetividade nas políticas de controle do consumo.

Destaca-se que, embora os marcos regulatórios sejam fundamentais para o controle do consumo de bebidas alcoólicas, sua aplicação isolada tem se mostrado insuficiente para enfrentar a complexidade do problema. É necessário reconhecer que o consumo de álcool está enraizado em práticas culturais, sociais e econômicas, exigindo, portanto, abordagens mais integradas e multidimensionais. A incorporação de estratégias educativas, comunitárias e intersetoriais - aliadas à regulação - pode ampliar o alcance e a efetividade das políticas públicas.

Entre os limites do estudo, destaca-se o delineamento transversal, com entrevistas telefônicas com adultos com telefone fixo, podendo não representar toda a população, contudo essa questão é minimizada pelo uso de fatores de ponderação dos dados. O indicador utilizado considera o consumo excessivo, que mede o consumo de bebidas alcoólicas em uma única ocasião de forma abusiva, comum em populações jovens e de maior escolaridade, em momentos de celebração, não necessariamente referindo-se a um consumo crônico, ou de dependência, o que é geralmente mais comum entre os mais pobres^25^.

Os resultados deste estudo evidenciam uma tendência preocupante de aumento do consumo abusivo de bebidas alcoólicas na população brasileira, com destaque para o aumento entre as mulheres, adultos em faixas etárias intermediárias e indivíduos com maior escolaridade. As variações regionais e entre capitais reforçam a importância de se considerar desigualdades territoriais e sociodemográficas no planejamento de ações de enfrentamento.

O cenário identificado indica que, mantidas as tendências atuais, as metas nacionais e globais de redução do consumo de álcool em 10% até 2030 dificilmente serão atingidas. Esses achados reforçam a urgência de se fortalecer políticas públicas abrangentes, que combinem medidas regulatórias, estratégias educativas, ações intersetoriais e abordagens sensíveis ao contexto social e cultural do consumo. Somente por meio de respostas integradas, sustentadas e baseadas em evidências será possível reverter essa tendência e promover ambientes mais saudáveis e equitativos para a população.
